# Supplementary material for: Identification of Genetic Polymorphisms of PI, PIII, and Exon 53 in the Acetyl-CoA Carboxylase-α (ACACα) Gene and Their Association with Milk Composition Traits of Najdi Sheep
Source: Animals (Basel). 2023 Apr 12;13(8):1317. doi: 10.3390/ani13081317 (PMC10135350; doi:10.3390/ani13081317)
Supplement: Supplementary file 1 [file animals-13-01317-s001.zip › animals-2291607-supplementary.pdf]

animals

Supplementary Material

Identification of genetic polymorphisms of PI, PIII, and exon53 in the acetyl-CoA carboxylase-α (ACACα) gene and their association with milk composition traits of Najdi sheep

Abdulkareem M. Matar*, Abdulrahman S. Alharthi, Moez. Ayadi, Maged A. Al-Garadi, Riyadh S. Aljummah

*** Correspondence:** Abdulkareem M. Matar: Abdmatar@ksu.edu.sa

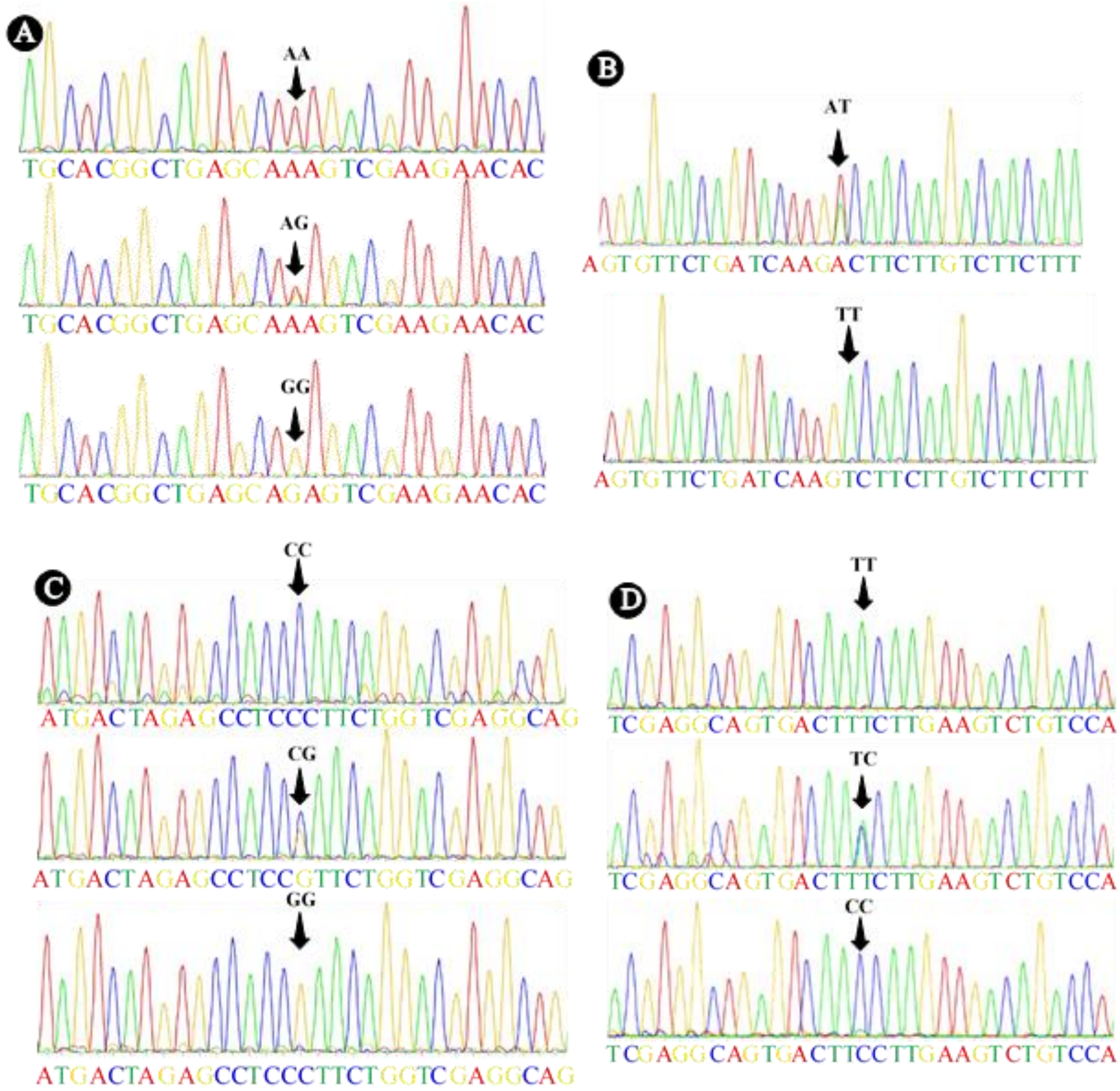

Figure S1. The SNPs identifying the promoter I region in the Najdi sheep *ACACα* gene as follows: **A**: 4412G>A; **B**:4441T>A, **C**: 4485C>G; **D**: 44507T>C.

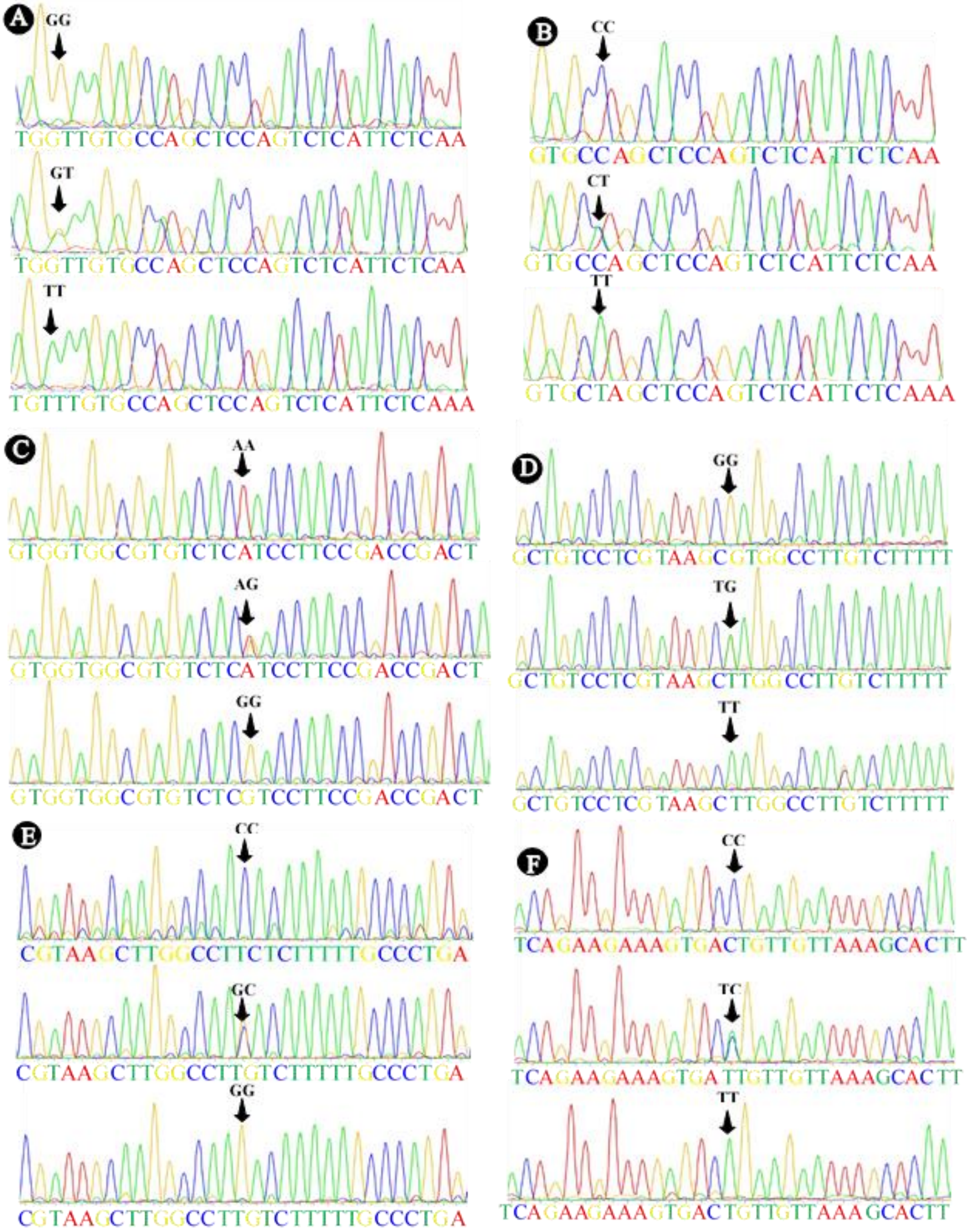

Figure S2. The SNPs identification of promoter III region in the Najdi sheep ACACα gene as follows: A:1007T>G; B:1014C>T, C:1168A>G; D:1331G>T; E:1339C>G; F:1431C>T.

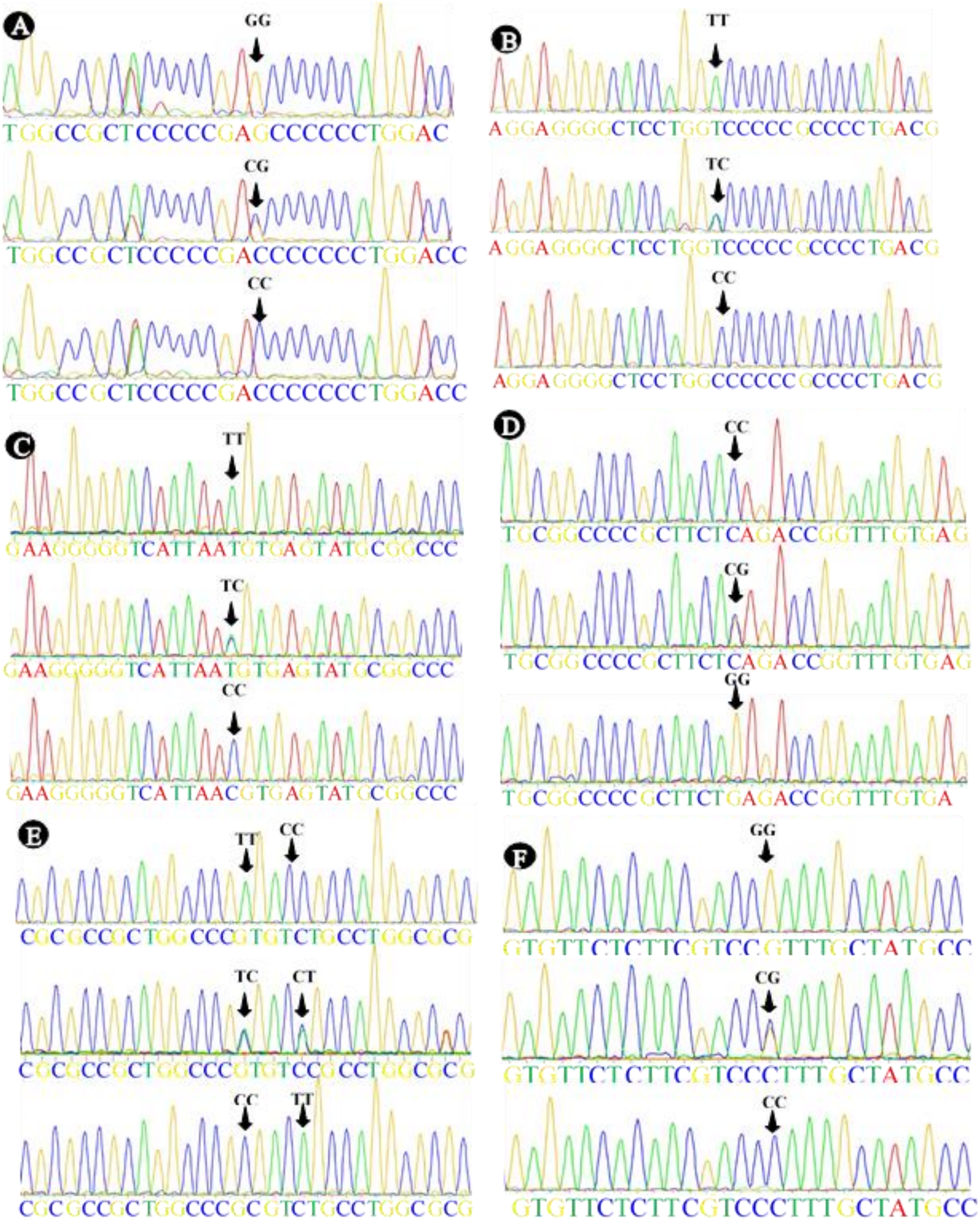

Figure S3. The SNPs identification of the exon 53 region in Najdi sheep ACACα gene as follows: A:6627G>C; B:6668C>T, C:6855T>C; D:6860G>C; E:(6894T>C and 6898C>T); F:6977C>G.

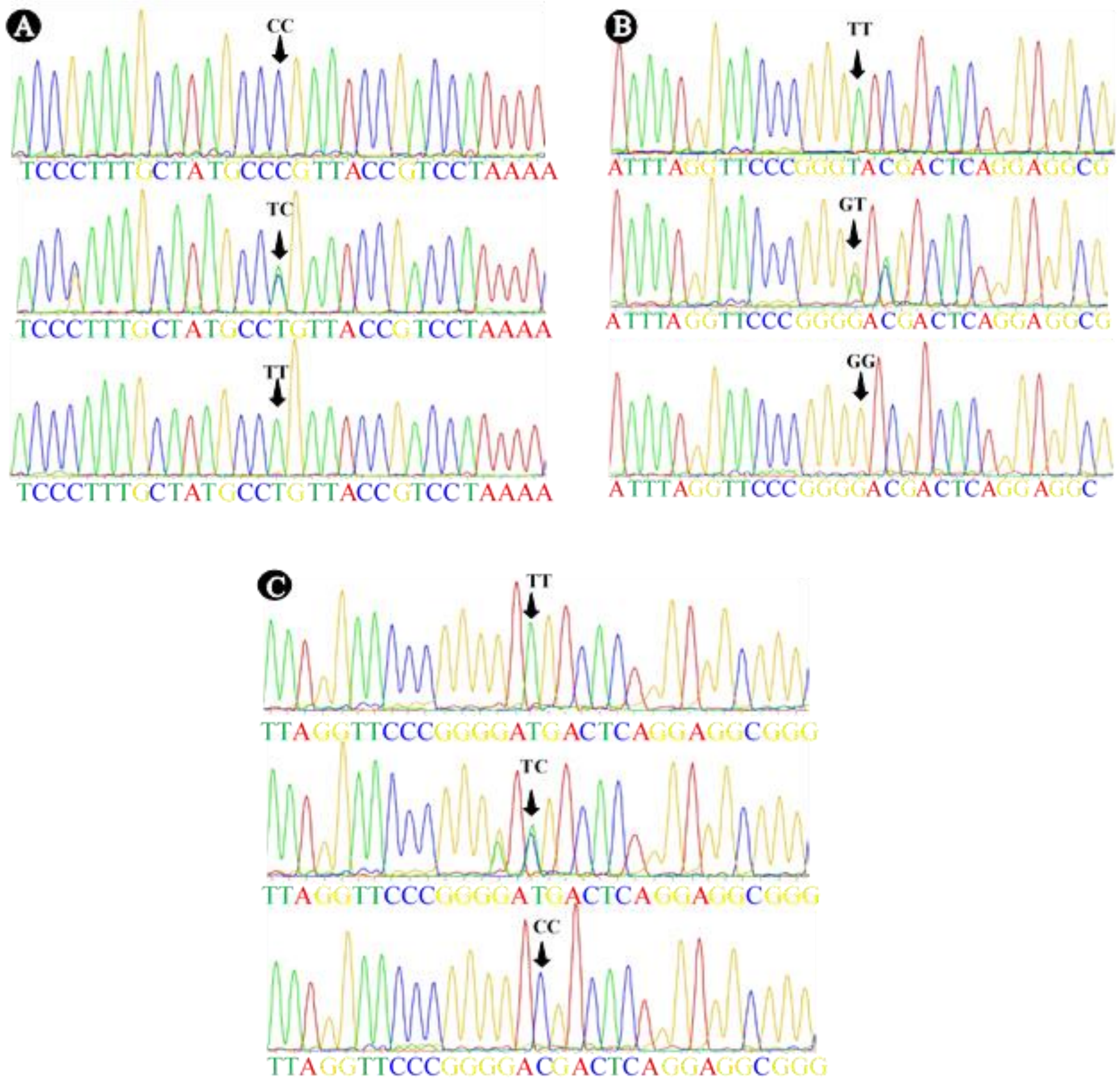

Figure S4. The SNPs identification of the exon 53 region in the Najdi sheep ACACα gene as follows: A:6989T>C; B:7029G>T, C:7031C>T.
